# Biological Role of Zinc in Liver Cirrhosis: An Updated Review

**DOI:** 10.3390/biomedicines11041094

**Published:** 2023-04-04

**Authors:** Muhammad Ikram Ullah, Ayman Ali Mohammed Alameen, Ziad H. Al-Oanzi, Lienda Bashier Eltayeb, Muhammad Atif, Muhammad Usman Munir, Hasan Ejaz

**Affiliations:** 1 Department of Clinical Laboratory Sciences, College of Applied Medical Sciences, Jouf University, Sakaka 72388, Aljouf, Saudi Arabia; aaalameen@ju.edu.sa (A.A.M.A.);; 2 Department of Medical Laboratory Sciences, College of Applied Medical Sciences, Prince Sattam Bin Abdul Aziz University, Al-Kharj 11942, Riyadh, Saudi Arabia; 3 Department of Pharmaceutical Chemistry, College of Pharmacy, Jouf University, Sakaka 72388, Aljouf, Saudi Arabia

**Keywords:** liver cirrhosis, zinc functions, metabolism, zinc deficiency, complications

## Abstract

Liver cirrhosis is a complication usually due to the consequence of persistent chronic liver disease. It is associated with different mechanisms, including hypoalbuminemia, impaired amino acid turnover, and micronutrient deficiencies. Consequently, cirrhotic patients can develop progressive complications like ascites, hepatic encephalopathy, and hepatocellular carcinoma. The liver is a vital organ that regulates the different metabolic pathways and transportation of trace elements. Zn is an indispensable micronutrient trace element involved in its crucial functions in cellular metabolic activity. Zn mediates its action by binding to a wide range of proteins; therefore, it imparts numerous biological effects, including cellular division, differentiation, and growth. It is also involved in critical processes for the biosynthesis of structural proteins and regulation of transcription factors and acts as a co-factor for the various enzymatic processes. As the liver is a significant regulator of Zn metabolism, its abnormalities lead to Zn deficiency, which has consequences on cellular, endocrine, immune, sensory, and skin dysfunctions. Conversely, Zn deficiency may modify the functions of hepatocytes and immune responses (acute phase protein production) in inflammatory liver diseases. This review has concisely stated the evolving indication of the critical role of Zn in biological processes and complications associated with liver cirrhosis pathogenesis due to Zn deficiency.

## 1. Introduction

Cirrhosis or end-stage liver disease, is the progressive form of scarring or fibrosis, resulting in loss of vascular and liver architecture. Chronic liver disease (CLD) is the main predisposing factor for fibrosis progression. Therefore, early fibrosis diagnosis could be an alleviating factor in the prompt management of cirrhosis [1,2] and cessation of complications. Fibrosis is the excessive growth of extracellular collagen, which results in constant wound healing for the hepatic injury [3]. In CLD, there is a progressive and long-lasting deficiency in the metabolism of hepatocellular tissue. The reactive oxygen species (ROS) group are responsible for oxidative stress on liver parenchyma cells and are liberated endogenously or outside the liver parenchymal cells [4]. 

Liver cirrhosis is the advanced stage of CLD, resulting in numerous metabolic complications due to hepatic dysfunction and portal hypertension, ultimately leading to hypoalbuminemia and liver cancer. The liver plays a crucial role in the metabolism of nutrients and the development of liver cirrhosis is as a consequence of defective metabolism, leading to poor prognosis or complications [5,6,7]. Micronutrients like zinc (Zn) have a crucial role in liver metabolism, and their deficiency leads to liver cirrhosis. Different mechanisms impede Zn deficiency. The binding of Zn with urinary proteins like alpha-2 (α-2) macroglobulin binds to Zn strongly [7] and causes the significantly increased excretion of Zn in urine [8]. Furthermore, impaired intestinal Zn absorption has also been documented in liver cirrhosis patients [9,10]. 

Liver cirrhosis is the 11th leading cause of death worldwide due to cirrhosis, with 1.32 million deaths in 2017 [11] and 2.4% global death reported due to this anomaly in 2019 [12]. A previous study conducted on the global burden of disease reported 112 million people with compensated cirrhosis, which shows 1395 cases of compensated cirrhosis in 100,000 population [13]. The death rate of cirrhotic patients is 10 per 100,000 in the United States [14]. Liver cirrhosis is the third most common cause of premature death in the United Kingdom [15]. Its incidence frequency has been growing faster than the frequently diagnosed cancer types like breast, bowel, lung, and prostate cancers [15]. Over 0.6 million CLD cases have been estimated in the United Kingdom, and about 10% of these cases are diagnosed with cirrhosis. Over 23% of people die from liver diseases each year, almost 400% higher than data from the 1970s [16].

The major etiologies of cirrhosis include viral and non-viral contributors. Viral hepatitis like hepatitis B virus (HBV) and hepatitis C virus (HCV) are the common factors, while liver steatosis caused by alcohol induction and non-alcoholic fatty liver disease (NAFLD) also contribute to cirrhosis development. Furthermore, inflammatory diseases like viral hepatitis can lead to chronic liver diseases. It is imperative to emphasize that from a bio-molecular level, the NS3 protease from HCV requires the Zn to bend into an active conformation and utilize its function to infect hepatic cells [17]. This is another indication that highlights the significance of Zn on liver homeostasis in health and disease and presents the intricacy between the relations recognized of this trace metal not only with human hepatic cells but also with exogenous virus particles.

**Figure 1:**
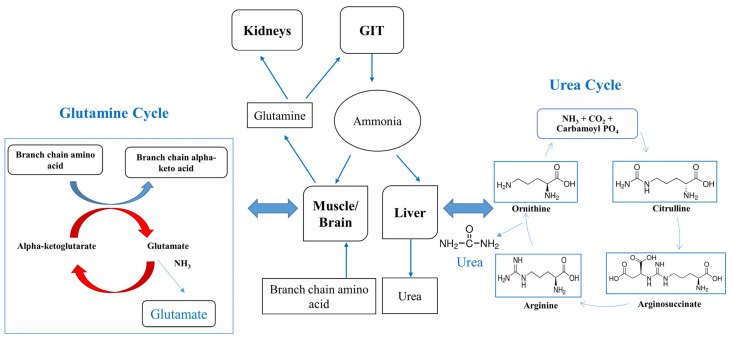
Outline of Ammonia Metabolism by Urea and Glutamine cycles in the Human body. The -glutamine cycle is a metabolic pathway that describes the formation of glutamate (reversible reaction-red arrow) from α-ketoglutarate carried out by glutamate synthetase, while branch chain amino acids are converted to branch chain α-keto acid by branch chain dehydrogenase enzyme.

In cirrhotic patients, hepatic encephalopathy (HE) is a frequent complication of neuropsychiatric phenotype. The pathogenic mechanism is not clearly explained, though the role of ammonia is stated extensively. Blood ammonia levels are found elevated in HE and liver cirrhosis in a frequency of about 90% of patients [18]. In liver cirrhosis, profound Zn deficiency is associated with HE [19]. Zn deficiency is linked to impaired nitrogen metabolism due to the reduced enzymatic activities in the urea cycle, like ornithine transcarbamylase in the liver [20,21,22] and glutamine synthetase in the muscle [21,22]. In liver diseases, including cirrhosis, the metabolism of ammonia is reduced. The utilization of ammonia production in the human body is carried out by the urea cycle (liver) and glutamine cycle (skeletal system) with a 1:1 ratio. In patients with Zn deficiency and chronic liver disease, inadequate ammonia clearance in the urea cycle lead to hyperammonemia. Hence the excessive concentration of ammonia is detoxified in the skeletal system by the glutamine cycle (Figure 1). Actually, the reverse relationship is documented between serum Zn levels and blood ammonia levels in liver cirrhosis; Zn decreases and ammonia increases [23]. 

The difference between free radical production and their neutralization leads to severe oxidative load on hepatocytes. It is responsible for the destruction of nucleotides for DNA synthesis, amino acids, and fats, leading to damage and apoptosis of hepatocytes. In addition, these reactive oxygen molecules are also discharged pro-inflammatory markers resulting in hepatic cirrhosis [24].

Trace elements are indispensable for a critical metabolism role and normal physiological functions. These trace elements are calcium, iron, magnesium, zinc, copper, manganese, cobalt, selenium, and molybdenum. These essential trace elements participate in different chemical reactions to execute the physiological function and homeostasis [25]. Alteration in the homeostasis of trace elements results in deficiency and toxicity of the specific trace element predisposing to disease pathophysiology. The liver regulates metabolic function in the human body and is involved in trace element transportation for tissue distribution, bioavailability, and toxicity level [24,26]. Trace elements participate as co-factors or prosthetic groups in many enzymatic reactions by triggering or inhibiting them, along with competing binding sites for metalloproteins and other elements. These are also crucial for maintaining vital functions, and loss of homeostasis (deficiency or toxicity) leads to metabolic ailments. As the metabolism of trace elements occurs in the liver, a substantial relationship is present between trace element metabolism, disease occurrence, and its progression [27]. 

## 2. Biological Functions of Zinc

**Figure 2:**
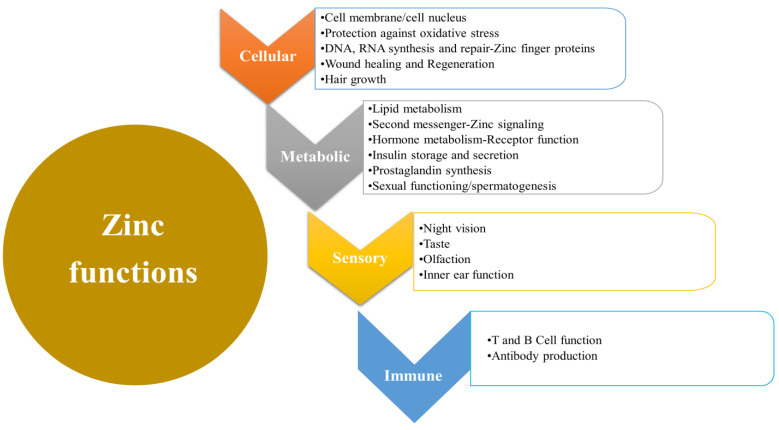
Biological functions of Zinc in the human body.

Zinc is an essential trace element with enormous biological functions, including cellular, metabolic, and immune functions (Figure 2). Zn functions as anti-inflammatory, antioxidant and apoptotic effects are well described [28]. It is mainly involved in cellular metabolism, specifically protein synthesis. In protein synthesis, Zn is a manipulator in the propagation of cells, diversity, and apoptotic function with dynamic expansion, revival, and cellular restoration. In the human proteome, the enzymatic catalytic domains carry zinc ions and the activity of zinc-binding proteins for more than 300 enzymes [29,30]. It also has a significant role in regulating immune functions at multiple reaction stages. In addition, it mediates its role in cellular growth and differentiation, gene expression, and metabolism [29,31]. The biological functions of Zn in human physiology are widely described, and its deficiency effects are predisposing in almost half of the world population [14]. A World Health Organization (WHO) report stated that Zn insufficiency is the fifth main risk factor for morbidity and mortality in poor or underdeveloped countries. Zn deficiency develops when its normal plasma or serum threshold level decreases from its lower normal limit of 100 µg/dL [32]. The effects of Zn deficiency can impart in different cellular and metabolic errors, including growth and oxidative depression, immune disturbances, and cognitive disorder. Due to its key biological association with protein activity, the enzymes are involved in the metabolism of collagen, consequently impacting the development of fibrosis [29].

## 3. Clinical Implications of Zinc Deficiency

The deficiency of zinc can lead to a variety of clinical expressions and is multifactorial. In chronic liver disease, changes in carbohydrate and lipid metabolism cause the precipitation of micronutrients and protein, resulting in malnutrition [33]. The clinical features include loss of or poor appetite, a change in taste and smell, body hair loss, delayed wound healing, and testicular dysfunction. It also causes decreased immunity and reduced drug elimination from the body [31]. 

The mechanism of action of Zn is mainly bound to proteins (albumin and alpha 2-macroglobulin) and acids which is directly proportional to the rate of Zn absorption. In progressive liver disease, the albumin level decreases, which may lead to reduced Zn absorption consequences in hepatic cellular carcinoma [31]. Other factors contributing to Zn deficiency in liver cirrhosis include decreased zinc absorption due to the inflammatory gut by cytokines (interleukin-6) and endotoxemia of the gut. This causes changes in the mucosa of GIT and decreases the absorption of Zn [34]. In addition, some drugs like diuretics increase the excretion of zinc and reduce the Zn binding capacity to albumin, resulting in Zn deficiency in the circulation. Furthermore, cirrhosis is complicated by hepatic encephalopathy (HE), ascites, variceal bleeding, toxicities, infections, and hepatocellular carcinoma (HCC). It characterizes the continuum of chronic liver diseases irrespective of etiology.

### Mechanism of Zinc Activity and Effects of Deficiency

Zinc participates in various physiological mechanisms of enzymatic reactions having a catalytic influence on the substrate exchange and maintaining the equilibrium of enzyme assembly. It also employs fundamental activities to control transcription factors and hormonal regulation by acting on the respective receptors of the hormone and for the specific gene function/expression. The role of Zn in metabolism and development has also been well-established [35]. Furthermore, it also entails a vital second messenger role, acts as a signaling ion, and disturbs redox activity. Zinc deficiency is the key source for the development of oxidative stress and cellular apoptosis [35,36]. While Zn^2+^ has antioxidant proper by having the redox indolent. The defense through-provoking vigorous redox conversion of metals such as iron and copper and the safety of the sulfhydryl protein group from oxidative injury is considered the most significant antioxidant source of Zn. Additionally, this element is significantly involved in the neurotransmitters’ metabolism, development, reproductive, thyroid, and insulin storage [36,37].

Zinc ions are not protein bounded and are required principally in intra-cellular parts of various cells [38]. It acts as a messenger in inter-cellular communication and the regulation of intracellular control [39]. Zn is one of the most abundant intra-cellular micronutrient elements found in all body tissues. The largest concentration of Zn is found in bones and muscles, which is about 85% of Zn stores, and the remaining 11% is present in the skin and liver. In an adult, 70 kg of weight is equivalent to 2–3 g of zinc [40]. In developed nations, the Zn concentration in the diet usually disbursed is normally sufficient to provide this requirement, and meat is a rich source of Zn [31]. 

Zinc is an essential co-factor for regulating diverse caspases, an enzymatic family comprising cysteine proteases that enterprises cellular apoptosis [41]. In addition, its regulatory role is established in molecular processes, intracellular signaling systems, and activation of growth factors, hormones, and cytokines [42]. Moreover, it is a proficient intra-cellular secondary messenger compared to the calcium regulatory pathway. The hindrance in the free mobility of intracellular ionic Zn^2+^ applies a critical influence on the progression and development of various diseases [43]. The intracellular homeostasis of Zn is imperiled as a protein regulator for numerous metabolic reactions. The presence of these types of switches accentuates Zn’s importance. Metallothioneins are Zn carrier proteins that act as key constituents of Zn absorption and storage in this pathway [44]. A set of discrete Zn transporters expedite the incursion and efflux of zinc through the cell membrane [45].

In human cells, zip transporters (Zip and SLC39 family) and zinc carriers (ZnT and SLC30 family) are present. ZnT condenses the intra-cellular accessibility of Zn by cellular efflux or into vesicles of the cell. Although, the Zip transporters enhance the intracellular trafficking of Zn by instigating its extracellular counterparts. Moreover, perhaps, it activates the discharge of Zn from the cytoplasmic vesicle [46]. Equally, both transporter types have a precise expression in each tissue with unlikely roles in controlling nutritional zinc insufficiency or superfluity and regulating the biological functions of cytokines and hormones. Additionally, different zinc transporters harmonize the cellular, sub-cellular, and organelle-based translocation of zinc ions [47].

Gene expression of cells is mediated by the proteins targeting the regulatory Zn^2+^ ions, curtailing the temporary zinc-binding sites on membrane receptors, the activity of enzymes, sensor proteins, and protein–protein interactions. The regulation of Zn through a series of processes relies on specific proteins that cause metal ions to be detached. In cellular activity, these findings support the essential Zn functions and its metalloproteins [44]. 

The expression of Zn carrier genes (*Zip1*, mRNA of ZnT) is exceedingly synchronized, which is independent of nutritional Zn [48]. The expression of ZnT mRNA is speckled substantially amid specific transporters. The most expressed transporters are *ZnT1*, *ZnT7*, and *Zip1*. Consequently, the enhanced expression of Zn transporter recycled as a substitute represents the intra-cellular Zn deficit, equivalent to ferritin consumption in iron deficiency. The Zn metabolism aberrations also disturb mitochondrial activities, resulting in DNA damage, developmental disorders, and congenital birth defects. The availability of Zn ions was demonstrated to be higher in an oxidative environment, whereas the reductive environment decreases its availability [46]. It is proposed that the Zn disruption of the antioxidant role in deficient cases may escalate the understanding of factors that cause DNA damage and reduce DNA repair mechanisms [49].

## 4. Metabolism of Zinc in the Liver

The liver mainly maintains the total body circulating Zn balance [50]. Any parenchymal change in the liver results in Zn deficiency, and zinc releases from hepatocytes are differentially controlled. Throughput discoveries expending that Zn^2+^ displayed comprehensive interchange of Zn in hepatocytes entails fewer than two days [51]. In lieu of metabolic activity, these constituents activate a momentary metabolic Zn dysregulation with ensuing plasma Zn scarcity. Some mediators and stress factors, identical to pro-inflammatory cytokines or lipo-polysaccharides, can have comparable properties [52]. Alteration in zinc status precisely disturbs the expression of gene/s. Perhaps, the expression levels of mRNA for metallothionein, retinol-binding protein, cholecystokinin, uroguanylin, endothelin, etc., increase or collapse in retort to variations in zinc concentration [53]. Lacking zinc marks the diverse hepatic roles and, having a significant role in metabolism, it also influences the metabolic developments in other organs [54].

There are constricted connections between Zn and metallothionein, an acute-phase protein primarily required for Zn absorption, distribution, and intracellular storage. Additionally, the higher zinc consumption generates a rise in metallothionein production. The adjacent communications between zinc and a cytokine, interleukin-6 (IL-6), have also been recognized, and IL-6 is significantly involved in regulating genes of acute-phase proteins [55,56].

The role of Zn in the liver also produces acute phase proteins, in the gluconeogenesis decree, in the volatile substrates (nitrogen-monoxide) mechanism or hydrophilic radicals, other than microbial growth control enhancing IL-6. IL-6 has a role in the *Zip14* transporter in the liver, promoting hypozincemia or low zinc with persistent acute-phase reactions [56]. The valuable characteristics of zinc transporters are also associated with hepatocytes; nevertheless, substantial breaches in indulgence persist. Thus far, IL-6 has leading activity for *ZnT5* (a bone growth factor) and has strong evidence for activities for *Zip5* and *Zip6* transporters (steroid hormones, prostate, and mammary gland). 

*Zip-14* is multifaceted in the induction of IL-6 of hypozincemia throughout provocative progressions. IL-6 interrelating with *Zip-14* shows similar impacts as in serum iron (hypo-ferritinemia) in inflammatory situations by modifying the hepcidin production in the liver [57], and *Zip-14* triggers the uptake of both zinc and non-transferrin bound iron (NTBI). In hemochromatosis, the higher NTBI concentration provokes surplus iron in different tissues predisposing to cardiomyopathy, diabetes, hepatocellular carcinoma (HCC), etc. In addition, it is suggested that the *HFE* gene causes hereditary hemochromatosis; *Zip-14* intervened in the transport of iron. The *Zip-14* transporter also intercedes zinc consumption through hepatic restoration [57]. In chronic liver disease, *Zip-14* mediating activity might epitomize a potential therapeutic target to endorse hepatic revival. Nonetheless, it also shows an intense role in the expansion of lumps, like in HCC. A report observed down-regulation of *Zip-14* reducing zinc in cancerous cells in HCC patients that augment tumor progression. It might be concluded that the *Zip-14* transporter is involved in the concentrated capacity of tumor cells to yield Zn [58].

## 5. Deficiency of Zinc in Liver Diseases

The deficiency of Zn has become an increasing medical or health issue worldwide. Zinc insufficiency was primarily recognized in 1960, and it is more frequent in all age groups and equally affects both genders. It may occur due to insufficient nutritional intake, diminished absorption in the small intestine, body requirements, less consumption of Zn and its loss through feces, urine, sweat, etc., and a variety of inherited disorders [59]. It is linked to various disorders, including chronic liver disease, malabsorption syndromes, and red cell pathologies. The most recent research by non-invasive high-throughput techniques [60] to detect nano-mechanical changes in the liver [61] or red blood cells [62], respectively, which may directly affect human pathologies. Various factors are responsible for developing Zn deficiency or can progress to alter the Zn metabolism in liver cirrhosis (Table 1). These factors include insufficient intake alterations in amino acid and protein metabolism, lessened portosystemic shunts, hepatic extraction, impaired absorption due to alcohol influence, and the effects of endotoxins and cytokines, primarily IL-6 [63,64].

There have been reports about severe muscular wastage resulting in considerable loss of Zn in the urine [65]. Due to the complications of ascites in cirrhotic patients, catabolism increases and demonstrates an immense fall in muscle activity. The diuretic treatment in cirrhotic patients and the complication of ascites marks not merely an augmented renal Zn elimination, nevertheless similar to condensing the serum albumin levels and compact volume of albumin to fix zinc [66].

In liver cirrhosis, several clinical characteristics have been linked to Zn deficiency, encompassing body hair loss, testicular atrophy, loss of or poor appetite, abnormal cerebral function, immune abnormalities, altered taste and smell, reduced or altered metabolism of protein, thyroid hormones, and vitamin A, late wound healing, and reduced drug elimination ability [67]. There is a wide variety of conceivable pathomechanisms of Zn deficit in liver cirrhosis. The deficiency of Zn may result in oxidative damage to tissues and/or the controlling influence on specific signaling systems in the liver [68]. The deficit of Zn may also be affecting oxidative pressure and ensuing environments like susceptibility to inflammatory hepatitis, loss of defense of acute phase in hepatitis, and factors for oxidation of lipids. In redox state variation, Zn deficiency supports transcription factors that are oxidatively favorable and can disrupt cellular function, dissemination, and existence [69,70]. This deficiency has variable impacts on liver activity, especially the restoration capability of the liver. 

Due to oxidative stress influence, reduced or deficit Zn can lead to cellular and tissue damage by modifying specific signaling cascades with subsequent configurational impairment of enzymes, mitochondria, and ribosomes [71]. Production of oxidative species stimulated by lacking Zn raises inflammatory parenchyma of the liver (hepatitis) and compromises the defense phenomena against viruses and lethal materials. The change in redox potential confines the reductive oxidation of transcription factors responsible for cellular activity diversity [70]. In liver diseases, oxidative tension may endorse gut penetrability with endotoxemia, infections like spontaneous peritonitis, and discharge of hormonal stress [71].

In addition, liver cirrhosis is linked with the reflective involvement of immunological abnormalities or their failures. For example, the reticuloendothelial system failure results in scarcity or low impact of immune reconnaissance characteristics, and decreased liver production of proteins complicates the innate immunity and forms the recognition site, delaying the capability of bacteria for the phagocytosis process [72,73,74,75].

### Zinc Deficiency in Liver Cirrhosis Complications

In liver cirrhosis, ascites is one of the most serious and life-threatening impediments. The liver shows a fundamental character in the directive of nourishment by governing the digestion of macro or micro-nutrients, their spreading, and apposite practice. Subsequently, protein-energy malnourishment in advanced liver disease patients characterizes a vital predictive marker for distressing mortality, the success of liver transplantation, and the value of lifespan [76,77,78]. In cirrhosis, the catabolic state has characteristic disproportionate plasma levels of amino acids: a decreasing or diminished branched amino acids (Isoleucine, Leucine, Valine) and excessive level of plasma aromatic amino acids (Phenylalanine, Tyrosine, Tryptophan) [79]. Moreover, amino acid and protein metabolism reorganization appeared by advanced undernourished muscle cessation. They lessened ammonia removal from muscles and the liver and muscles, and cirrhotic patients suffered from metabolism turbulences with better energy disbursement and repeatedly improved hyperactive catabolic rate [80].

Several reports have described that supplementing some basic amino acids or with a zinc mixture might develop hypo-albuminemia and ascites, causing the amplified source of substrate for proteins and the incentive of protein combination [81,82,83]. Albumin has been revealed to be a multi-functional protein with antioxidant, immune modulator, and reclamation purposes [84]. It shows an ultimate character in the metabolites dissemination; Zn and copper ion transition, hormones, and drugs [85]. Albumin is a main transport protein of Zn in the plasma, and about 80% of plasma Zn is transported by this mode [86]. It has been described that liver cirrhosis is linked to massive Zn deficiency, which progresses to abnormalities of nitrogen metabolism [87]. The branched change amino acids are twisted in innumerable biological progressions like stimulus of albumin and glycogenesis, upgrading of insulin confrontation, stimulation of mitochondrial bioenergetics, limitation of ROS production and apoptosis of hepatic cells, and improvement in liver restoration [88]. In randomized clinical trials, it has been presented as a conceivable outcome of branch chain amino acids (BCAAs) in the control of CLD [89]. Two mechanisms have been involved in reducing hypoalbuminemia in regard to the ability of BCAAs rich supplement.

Additionally, another report described that constant nutritional supplementation with BCAAs prompted ribosomal phosphorylation of the S6 protein in the liver of rats with CLD. Therefore, subsequent higher production of albumin BCAAs supplementation can increase an osmotic pressure, which results in extracellular fluid reduction and can lead to ascites progression. The nutritional supply of Zn might affect the depletion of BCAAs in muscle mass. Therefore, the intake of BCAAs might be utilized for albumin synthesis, which reduces serum concentrations of albumin and results in the reduction of ascites degree [89]. Zinc stored in different organs like the liver, muscle, and bone can be replaced independently of the severity of liver cirrhosis and complications such as diabetes mellitus.

In another study, children suffering from malnutrition with chromosomal damage and cytotoxic properties could be restored in vitro with Zn sulfate nutrients. Nonetheless, it should be known that more investigations involving large patient numbers are required to govern the comprehensive application of achieving and maintaining clinical productivity in liver cirrhosis management [90].

Hepatic encephalopathy (HE) is a reversible neuro-psychiatric syndrome that might emerge in serious disorders like acute and chronic liver conditions. Approximately 70% of cirrhotic patients are involved in incidences of HE. While the pathogenesis of HE is diverse and heterogeneous, the higher concentrations of ammonia contribute a critical role in this pathway. Hyperammonemia persuades the inflammation of the astrocyte cells and concomitant deviations that dislocate neuronal communication and decrease the brain’s energy provision. In addition, edema in the astrocytes influences the fundamental brain proteins and activates nitrosative and oxidative pressure [91,92]. 

**Figure 3:**
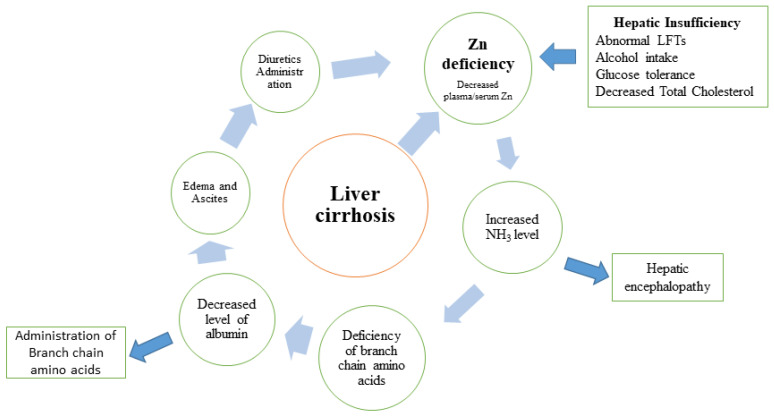
Zinc deficiency and liver cirrhosis.

Further aspects of excessive ammonia that are complicated in the HE pathogenesis contain contagions, abnormal neutrophil functions, and the properties of pro-inflammatory cytokines [93]. The poor status of Zn spoils the metabolism of nitrogen by tumbling the action of enzymes of the urea cycle in the liver [10] and in the skeletal muscle [79]. Zn deficiency is linked with metabolic nitrogen alterations, in an investigational rat model of cirrhosis and progressive liver disease patients [94]. It is proposed that the diminished Zn may be involved in the HE pathogenesis, as serum Zn levels are curtailed in HE patients and associated inversely with ammonia concentrations in the blood [95]. The mechanism is presented in Figure 3. It is also supported in another report of a patient with severe HE and a Zn deficit [96]. The longer duration of Zn supplementation enhanced the improvement in encephalopathy and eminence of lifespan by depressing the circulating ammonia. Very few controlled studies have commenced scrutinizing Zn proficiency for treating HE cases associated with liver cirrhosis [97]. Overall, some clinical-oriented controlled reports of Zn replacement in HE patients endured minor and inconsistent outcomes. One of the fascinating statements suggests that the exchange of Zn has a valuable result on the muscle spasms commonly agonized by liver cirrhosis patients [98].

Another complication of liver cirrhosis, hepatocellular carcinoma (HCC), is considered the fifth most common type and third most death-associated cancer globally [99]. Liver cirrhosis is the conclusive disease risk factor causing the development of HCC. The mechanism of hepatic carcinogenesis is not clearly understood; however, exclusive details have been explicated. It is understood that the involvement of signal transduction paths alleviated cancer progression and may represent the objective constructions for systemic recovery. Still, there is massive HCC heterogeneity, and the wide-ranging energies are necessary to express the molecular genesis of HCC, evidently [100]. 

The close relationship between chronic inflammatory effects and cancer expansion has been clearly documented. HCC cases increase due to the genetic variability associated with chronic inflammation exposure. The inflammatory factors may be diverse, comprising viral infections and toxic sources like alcohol and aflatoxin. Moreover, metabolic instabilities or syndromes, like diabetes mellitus, may persuade and disseminate chronic inflammatory responses, stimulating cancer expansion. Many heterogeneous regulatory agents and signal pathways are concerned with the carcinogenesis elicited by infection. In the cellular aging mechanism, chronic inflammation results in early cellular death, telomeres limitation, and genetic uncertainty. The relationship between chronic inflammation exposure and immunological deviations controlled by viruses can be evidently described in hepatitis C predisposition [101]. 

HCV-induced infections carry immunological variations that devastate the hepatocytes, whereas generating recreating routes at the same point maintains the inflammation, affects the oxidative stress, and may damage the DNA. At this stage, HCC seems to develop obstinate inflammation with progressive fibrosis, ultimately progressing to liver growth [99]. Zinc is one of the essential trace elements with diverse functions, and its low concentrations in plasma are associated with HCC. Plasma zinc levels are exaggerated by anxiety, impurities, disturbances, and malicious routes. The raised serum copper levels are a decree linked with reduced zinc meditations [100], and a connection between high serum copper and possible pathogenesis of HCC progression has been established. Conversely, the zinc concentration was abnormally low in HCC tissue compared to its level in the healthy liver parenchyma. A retrospective analysis of liver cirrhosis patients presented about 18.5% of cases of HCC. Of these patients, about 76.7% displayed zinc deficiency. About 55% of alcohol-induced cirrhosis cases unveiled decreased zinc levels, and zinc deficiency was detected in 95% of chronic hepatitis C [101]. It is not yet clear whether the fluctuations of zinc levels between serum and tissue subsidize the tumor development or are, as an alternative, influencing the malignant transformation of the situation.

Depending on remarks of intra-cellular Zn levels in dissimilar cancers, it has been hypothesized that these aberrations may lead to tumor progress by impressing Zn signal function, which ensures the inhibitory or triggering possessions on a wide variability of molecular assemblies. Furthermore, there are receptors, transcription factors, and caspase kinases, and phosphates. A fundamental part possibly follows abnormalities in the expression of different zinc transporters (Zip, ZnT), as it has been recommended in the initial research on certain measures of pancreatic and mammary carcinoma [51]. A previous study detected a reduction in the Zip14 expression profile, fixed with a lessening of zinc concentrations throughout the change and evolution of HCC [70,102].

## 6. Conclusions

The biological properties of zinc have been illustrated widely. The roles of zinc are stated in organic compounds metabolism (Glucose, Protein, and Fat), immune functions, and hepatocarcinogenesis. Zinc deficiency reduces the capacity of the liver to metabolize appropriately, resulting in impaired protein metabolism. This phenomenon significantly contributes to the development of chronic liver disease and cirrhosis, proposing the efficiency of zinc supplementation therapy. Advance studies would be necessary to explain whether zinc transporters in hepatocytes have the restraint or prompting influence on the zinc-dependent signal cascades that are substantial in the growth of hepatocellular carcinogenesis. The contribution of zinc to pathologic conditions other than impaired nitrogen metabolism in patients with liver cirrhosis and possibilities for treatment also requires further study. While HCC’s huge heterogeneity may control only a small patient group, it may still represent a precise, thrilling, and esteemed assignment. It would take longer to understand tailored, personalized medicine perception.

## Figures and Tables

**Table 1:** Zinc deficiency and associated conditions in liver diseases [59].

Causes of Liver Disease	Mechanism
Insufficient nutritional consumption	Protein and amino acids metabolism variabilities
Contracted hepatic abstraction	Porto-systemic shunts
Alcohol-induced defective absorption	Production of cytokines, primarily interleukin-6 (IL-6) Production of Endotoxins by Biological pathogens

## Data Availability

All the available data included in this study can be found in the text of the manuscript.
